# A Novel Disability Advocacy and Awareness Program for Training Future Healthcare Professionals on Care for Patients with Disabilities

**DOI:** 10.7759/cureus.33881

**Published:** 2023-01-17

**Authors:** Priscilla R Morelli, Shemar Crawford, Vivian Tanios, Stephen Chelko, Alexandra Nowakowski

**Affiliations:** 1 Medical Education, Florida State University College of Medicine, Tallahassee, USA; 2 Biomedical Sciences, Florida State University College of Medicine, Tallahassee, USA; 3 Geriatrics, Florida State University College of Medicine, Orlando, USA

**Keywords:** patient-centered care, disability advocacy, disability training, inequity, health disparities, disability care, disability education, medical education

## Abstract

In a poll of 714 US physicians, it was revealed that only 40.7% felt very confident in their ability to provide the same quality of care, overall, to patients with disabilities (PWDs) compared with patients without disabilities. It was also found that only 56.5% strongly agreed that they welcomed PWDs into their practice as healthcare providers. This suggests a systemic issue of inequity in medicine, which affects both physicians and patients. If this problem is not corrected, our healthcare system will continue to lack in providing adequate care to PWDs. A key component of this issue is that the lack of confident care for PWDs appears to be a result of insufficient exposure to PWDs during the formative years in medical schools. Although medical students are taught extensive clinical skills and bedside manners, there appears to be little mention of how to make adaptations to basic patient encounters to accommodate PWDs. Further, the lack of representation of PWDs in the medical community results in minimal experience among trainees and the perpetuation of unjust biases in the healthcare system. Changes to the medical field must start with shaping future physicians and filling the void in medical education. As a solution, we at Florida State University (FSU) College of Medicine (COM) propose a program called the Disability Advocacy and Awareness Program (DAAP). Two interactive sessions were designed, and students were offered an immersive experience in which they were not only provided with information through well-crafted presentations but also encouraged to engage in direct interactions with PWDs and a myriad of assistive devices. We believe a great deal of the program’s success stemmed from the two-phase interactive model that allowed students to undergo a truly immersive experience that a textbook cannot endow. Although we cannot expect every provider to be an expert on all disabilities, all physicians should have an understanding of how a disability may impact a patient’s life and medical care. Improved knowledge and awareness surrounding disability and the barriers faced by the PWD population will provide insights that will allow for the most equitable, patient-centered care for the disabled community.

## Introduction

It has been reported in the literature that about 5% of medical students have some form of mental, physical, or sensory disability [1]. According to the Center for Disease Control (CDC), 26% of the United States population are living with a disability [2]. Despite the prevalence of disabilities within the medical community and the general population, few medical schools have instituted a curriculum to educate students on how to treat patients with disabilities (PWD). A survey of 75 medical schools revealed that only 52% of schools had some form of disability education as part of their curriculum [3]. A poll with 714 physicians revealed that only 40.7% felt very confident in their ability to provide the same quality of care overall to PWDs compared with patients without disabilities and that only 56% strongly agreed that they welcomed PWDs into their practice as healthcare providers [4]. These data suggest a systemic issue of inequity in the field of medicine, disadvantaging PWDs as both physicians and patients. If the root of this problem is not addressed, our healthcare system will continue to fall short of providing adequate care to PWD.

A key rationale behind the lack of confident care for PWDs is the insufficient exposure of medical students to this population during the formative years in medical schools. For example, physicians, residents, and medical students have shown a lack of knowledge (based on disabled patients’ perspectives) for commonplace disabilities such as cerebral palsy and learning disabilities [5]. PWDs report facing negative attitudes and behaviors, creating an inhospitable environment for them, which is a barrier to the provision of adequate care [2]. Not only is there a lack of access to healthcare, but there is also a lack of quality care. In the United States, a survey of approximately 600 PWDs revealed that only 44% felt that their healthcare providers were trained to provide general services to individuals with their type of disability and 61% indicated that their healthcare providers could benefit from more training [6]. The effect on the quality of care may stem from the lack of exposure. Two systematic reviews showed that when there is an increase in exposure, providers with more experience with PWDs had more positive attitudes with less anxiety or fear while providing healthcare services to this population [7,8]. For the purposes of brevity, we refer to individuals in the disability community using person-first language. We recognize that individual preferences do vary and some may prefer identity-first language or other terminology [9]. 

We at Florida State University (FSU) College of Medicine (COM) propose the initiation of a program called the Disability Advocacy and Awareness Program (DAAP). In this two-phase program, we would engage students in interactive sessions involving disability devices and scholarly speakers who are experts in the field of disabilities or have first-hand experience with PWD. The DAAP had many objectives, but the two overarching themes were (1) to provide a framework that clinicians can use to engage in respectful and meaningful clinical interactions with PWDs and (2) to promote the philosophy that the physician-patient interaction needs to involve compromise and negotiation toward a shared goal that is both realistic for the patient and conforms to the highest standards of patient care from the provider. The objectives of this program were adapted from *Core Competencies on Disability for Health Care Education* [10]. In this paper, we outlined how this two-phase program was developed to edify students through a multidisciplinary approach, which we hope will serve as a guide for other institutions hoping to instill these practices in their medical students in a similar fashion.

## Technical report

Program Overview

Two interactive sessions were designed and offered to students in an immersive experience that provided not only information via well-crafted presentations but also interactions with PWD, along with a myriad of assistive devices. In this manner, we aimed to give students a deeper understanding of disability etiquette, Americans with Disabilities Act (ADA) compliance, disability devices, and healthcare access for PWD. The complete list of objectives adapted from Core Competencies on Disability for Health Care Education for the program can be found in Table 1 [10]. We believe these concepts cannot be obtained or retained from simply reading text. As such, our DAAP sought to provide an experience to cultivate empathy and compassion in future healthcare providers. Since a major source of hesitation among providers appears to be rooted in a lack of exposure to PWD, we prioritized including interactive components with active student participation in the program. This stimulates students to interact at their own comfort level. We provided didactic introductions prior to each interactive session because we wanted students to feel as prepared as possible for each new experience.

**Table 1:** Program objectives*. *Adapted from *Core Competencies on Disability for Health Care Education *[10]

Overall program objectives	
1.	To explore and mitigate one’s own implicit biases and avoid making assumptions about a person’s abilities or lack of abilities and lifestyle
2.	To treat all patients, regardless of disability and functional status, with respect and humility
Phase 1 objectives	
1.1	To instill the right for PWDs to equitable access to appropriate, accessible, and high-quality healthcare
1.2	To define disability as a functional limitation and identify disability prevalence, as well as To discuss the diversity and range of disabilities
1.3	To describe how social determinants of health directly impact PWDs (e.g., discrimination, employment, poverty, and lack of access to education, transportation, housing, and healthcare)
1.4	To identify basic requirements of the Americans with Disabilities Act (ADA) applicable to healthcare and discuss strategies for meeting these requirements
1.5	To understand that PWDs may consider their devices to be an extension of their person and interact with said equipment in a polite and professional manner only after consulting with and obtaining consent from the patient
1.6	To describe various models of team approaches when supporting PWDs in healthcare systems (e.g., interdisciplinary, multidisciplinary, and/or inter-professional)
1.7	To describe the importance of teams and discipline-specific responsibilities of team members in addressing the healthcare needs of PWDs and recognize the necessity of incorporating the patient as a central member of the team
Phase 2 objectives	
2.1	To understand that PWDs should be the primary source of information regarding their care
2.2	To provide patient-centered care by creating a respectful environment in which a patient’s preferred identity and vernacular regarding disability etiquette are considered
2.3	To prepare providers to recognize their own biases and need for further skill development in caring for PWDs and to take actions to address these needs based on current best practices
2.4	To recognize that PWDs experience the same common health conditions as people without disabilities and that a disability may or may not impact the presenting signs and symptoms
2.5	To establish the importance of the knowledge that all interactions between the physician and PWDs need to revolve around compromise and negotiation toward a shared goal that is both realistic and conforms to the highest standards of patient care.
2.6	To not only understand that the same physical exam maneuvers can be performed on patients with or without disabilities but also that modifications need only be made based on communication with patients regarding their individual needs and abilities
2.7	To demonstrate an understanding of the skills required to perform a history and physical exam (PE), making modifications as needed to provide equal and effective care while accommodating for mobility, sensory, cognitive, and/or behavioral issues in the patients
2.8	To explore and mitigate one’s own implicit biases and avoid making assumptions about a person’s abilities or lack of abilities and lifestyle.
2.9	To demonstrate through first-person experiences that PWDs are typically knowledgeable of their own condition and that this expertise should be respected and used to improve healthcare decisions and care

Methods

Planning and Recruitment

This study (titled Need for Disability Training in Medical Education Curricula) was approved by the FSU’s institutional review board (IRB; Study No. STUDY00002837 to P.M.). The study was reviewed by FSU IRB and was deemed exempt.

A team of three medical students at different educational levels and two faculty advisors led the planning and implementation of the DAAP. Initial planning was conducted on several Zoom meetings with guidance from other faculty members, mentors, and advisors in support of this endeavor. Once the schedule of events (found below) was drafted, the students enlisted the assistance of registered student organizations (RSOs) to obtain funding and sponsorship for the sessions. Volunteers from FSUCOM and the Tallahassee community were then selected and recruited to serve as speakers and table hosts based on their areas of expertise.

To recruit student participants, a flyer was designed and distributed via emails among first- and second-year medical doctors and physician assistants at FSUCOM. These students were sent a Qualtrics survey through which they could sign up for the events. They were also notified in this email that those who attended both sessions would be awarded a certificate of attendance to further encourage participation. Of the 370 students who received the email, 26 completed the interest form and 25 participated in both events, as indicated by the attendance form used during phase I and the participants list on Zoom for phase II.

Schedule of Events 

The DAAP took place in two phases that were hosted on two consecutive Saturday mornings from 8 A.M. - 12 P.M. EST at the FSUCOM main campus in Tallahassee, Florida. Phase II also included a Zoom option to accommodate and protect our patient panelists and participants from COVID-19 infection. Due to the highly interactive component and hands-on learning necessary to achieve the objectives of phase I, no Zoom option was offered. Students and volunteers participating acted in accordance with the COVID-19 guidelines outlined by the CDC at the time. The schedule of events for the DAAP can be found in Table 2.

**Table 2:** Schedule of events in the DAAP.

Phase I	
8:00 AM - 8:30 AM	Student check-in, breakfast, introductions
8:30 AM - 9:15 AM	Recorded lecture: “Intro to Disability”
9:15 AM - 9:45 AM	Recorded lecture: “Accommodations and the ADA”; student-led discussions
9:45 AM - 10:00 AM	15-minute break
10:00 AM - 11:45 AM	Interactive assistive devices session (list provided below)
11:45 AM - 12:00 PM	Wrap-up and closing remarks
Phase II	
8:00 AM - 8:30 AM	Student check-in, breakfast, introductions
8:30 AM - 10:00 AM	Live lecture: Patient-Provider Communication and Disability Vocabulary
10:00 AM - 10:05 AM	Break
10:05 AM - 11:55 AM	Patient panel discussion and question/answer session
11:55 AM - 12:00 PM	Closing remarks

Phase I

Overview

Phase I of this series included a series of lectures focused on defining disability, types of disabilities, accessibility, biases, stigmatization, and mistreatment for those with varying disabilities. Another lecture examined what the ADA is and how accommodations play a role in the healthcare system and society as a whole. Both of these recorded lectures were made available to students in a follow-up email after the conclusion of the program. Following the lectures, students were given a 15-minute break, during which snacks were provided, and then rotated among seven device stations so that they learned about the different assistive devices associated with a wide range of disabilities. Each station was led by professionals in their respective fields, including physical therapists, audiologists, speech-language pathologists and instructors, occupational therapists, and others (see Acknowledgements). This interactive and stimulating event allowed students to get a better understanding of how assistive devices work from the unique perspective of different professionals.

In addition, the students had the privilege of using these various devices with the help of instructions from respective professionals. These stations are discussed below in detail. The primary goal of this engagement was to give future physicians insights into what their patients may use to thrive in their everyday lives. We believe physicians in every specialty should have a basic understanding of the most common assistive devices used by people with disabilities to be better educated about PWDs and their chronic illnesses and care for these patients. This is especially true for primary care physicians who serve at the frontline of medical care and may be the first ones to get the opportunity to correct improper use and improve quality of life or prevent an injury. By bringing in professionals from other medical fields such as those indicated in Table 3, we sought to cultivate an awareness of interprofessional networks that can benefit all patients and healthcare providers. With a better understanding of what other professionals do and how their fields serve as integral parts of the medical team, we believe physicians will be better prepared to make proper referrals and obtain the assistance of these professionals in patient care. We believe our approach will encourage future physicians to be more skilled at helping patients who use assistive devices or creating disability-friendly accommodations in their clinics. 

Didactic Session

For the first half of phase I, two lectures were presented to students. An FSUCOM associate professor first gave a presentation where he defined disability, clarified the medical vs. social models of disability, introduced the different types of disabilities, and discussed biases in society. The presentation challenged students to view disability from a different perspective and better understand the impact of stigma and social norms on the disabled community. An FSUCOM counselor and ADA representative then provided a brief overview of the ADA and associated laws and addressed obstacles within the healthcare system for PWDs. A student-led discussion followed the presentation to answer questions and encourage students to brainstorm examples of obstacles faced by PWDs in and out of a healthcare setting as well as solutions to these obstacles. The conversation then turned to assistive devices and the ways they may help PWDs not only live a happy and independent life in their community but also gain better access to medical care.

Interactive Session

The atrium of the FSUCOM was set up with seven stations for students to rotate through. In groups of four or five, students rotated through the stations after every 15 minutes. At each station, professionals described their field and role in patient care. These professionals then explained and demonstrated the assistive devices they use or provide to patients in practice. Before moving on to the next station, students were given the opportunity to use these devices under the supervision and guidance of the table hosts. Each table host was given the objectives in advance so that they could choose how to instruct students in a way that works best for their tools and area of expertise. An outline of the stations, professionals, and assistive devices can be found in Table 3.

**Table 3:** Interactive assistive devices in different stations.

Station number	Station name	Assistive devices/services presented
1	Physical therapy	Canes, walkers, and rehabilitation
2	Physical therapy	Crutches, manual wheelchairs, and bracing
3	Occupational therapy	Adaptive eating/cooking utensils and tools for strength training and activities of daily living.
4	Audiology	Various hearing aids and other hearing technology
5	Speech-language pathology	Communication aid interfaces and language adaptive technology
6	Vehicle modification	Multi-function display and a local resident’s personal vehicle
7	Power wheelchairs	Custom and standard power wheelchairs, customization options, seat transfer technology, and supportive seating

Phase II

Overview

Phase II of this program focused on providing students with a direct experience with PWDs and improving communication between future physicians and the PWD population. Through a lecture specifically created to educate healthcare providers, students were given a preface for how patient-provider interactions should be adapted to provide the best care to PWDs. We then transitioned to a question-and-answer style panel consisting of PWDs from the local community so that they could speak about their experiences with the healthcare system. It was our goal to provide students with the tools needed to engage in proper interaction with PWDs. We believe that hearing the lived experiences of PWDs directly from them would have a larger impact on the students than simply facilitating learning about protocols in a classroom setting.

Didactic Session

Phase II began with a lecture denoting the proper communication etiquette for PWDs with an emphasis on the patient-provider relationship. FSU professionals in communication science and disorders, augmentative and alternative communication, communication development, linguistics, and more presented an in-depth virtual lecture on proper communication techniques and an understanding of the science behind communication. Students were introduced to both the social and medical models of disability and taught the differences between person-first and identity-first language. Like in any other encounter, the physician’s word choice and accommodations toward the patient's circumstances (i.e., disability in this case) should be taken into consideration. Students learned about the ADEPT-CARE protocol (Table 4), which was developed by medical students and faculty to give medical providers in training a nine-step guide for assessing the healthcare needs of PWDs [11].

**Table 4:** ADEPT-CARE protocol. Protocol developed by medical students and faculty to give medical providers in training a nine-step guide for assessing the healthcare needs of PWDs [11]

ADEPT-CARE acronym	Description
A	Ask about access needs and accommodations in the healthcare environment
D	Defer to the disabled person
E	Engage with the patient
P	Promote participation and patient-centered care
T	Take time for a thorough medical history and PE
C	Consider disability-related conditions
A	Ask about access needs and accommodations in the home and community
R	Review the treatment plan and respond to feedback
E	Ensure accessible follow-up and referral

Interactive Session

A patient panel composed of three patients with different disabilities spoke about their unique experiences in the field of medicine. They discussed the circumstances that shaped their lives, which provided students with a better understanding of how such obstacles can be overcome and what “normal” looks like for PWDs. By hearing about these experiences, we hoped that students would be desensitized to disabilities and gain insights into how PWDs perceived their own circumstances. For example, by hearing a PWD refer to their daily activities as a normal part of life rather than an obstacle to overcome, we wished that these future healthcare professionals would stop using terms such as “inspiring” when referring to a disabled patient, thereby bringing an end to such insensitive rhetoric. In addition, the patients provided examples of exemplary vs. poor healthcare interactions. This included poor and/or insensitive physical exam (PE) practices, dismissal of patient concerns, assumptions based on patients’ disabilities, and disrespect of patient autonomy. All three panelists mentioned the importance of partnership with physicians. In this manner, students gained a deeper understanding of how to properly address patients while maintaining their dignity and respect. The patient panel also commented on how PEs could be altered and improved for future physician implementation. Through this workshop, our students could get a diverse viewpoint on how to respectfully treat all patients according to their needs.

## Discussion

Although we do not expect every provider to be an expert on every disability, all physicians should have an understanding of how a disability may impact a patient’s life and medical care. Additionally, medical professionals should have the compassion necessary to be mindful of biases and avoid the perpetuation of adversity in healthcare. By undertaking two interactive workshops, students were able to see a different side of disabilities and medicine. How the workshops were organized played a crucial role in their success. Although this article focuses primarily on the design of the disability curriculum workshops, it is important to note whether students found these workshops beneficial. 

Limitations of this project included the following: the inclusion of a small number of student participants, a lack of quantifiable results through surveys, and complications related to COVID-19. Although all first- and second-year medical and physician assistant students at FSUCOM were invited to sign up for the study, only 25 students participated. We believe this was due to multiple factors, including the timing of the events on Saturday mornings and the constraints of the academic calendar due to the fact that the events were conducted quite close to the holidays. The next program is intended to be conducted as three shorter sessions in order to reduce the time commitment for each day. Two of the three sessions will be hosted on weekday evenings, which students preferred over weekend mornings. The last event will be hosted on a Saturday morning to accommodate the assistive devices professionals’ schedules, but the session will be shorter than this program’s session to encourage increased participation. Greater efforts will be made in the future to advertise this program to students. We plan to inform students of the event earlier, promote it on social media platforms, and include it in the COM’s weekly newsletter. In addition, surveys will be created for the next iteration of this program to test students’ knowledge and comfort surrounding the topics included in the program. This survey will be distributed to student participants before the first session and after the final session to quantify changes in student perspective and support the success of this program. If pandemic-related guidelines permit, we would also like to have a patient panelist present in person for future events to allow students to have a more interactive experience with PWDs. Although we plan to continue offering a Zoom option as appropriate to be inclusive of at-risk individuals, we would like to have an immersive experience for those participants who are able and comfortable being physically present. 

The aim of phase I was to increase students’ knowledge of assistive devices (i.e., their implications and proper use) so that they feel comfortable teaching patients how to use them. By allowing students to use assistive devices with instructions from respective professionals, such as physical therapists and audiologists, they could gain a deeper understanding that lectures alone would certainly have not provided. Furthermore, they learned more about the roles these different professionals play in the medical team and how they can assist to provide a level of care that takes into account all aspects of a patient’s life. Not only did students enjoy this interactive learning activity but the professionals we invited to attend were also very grateful for the opportunity to participate in this program. All the professionals recruited expressed their disappointment with how little their skills and competencies are utilized by physicians. They feel under-consulted and that clinicians do not have a good understanding of the services these fields can provide to improve a patient’s life. By being able to present to actively engaged students, these professionals feel they were able to change this narrative and improve not only the utilization of their practices and interprofessional teamwork but also future patient outcomes. 

Our overarching goal with phase II was to increase the number of students who would feel as comfortable performing a complete PE on a PWD as they do performing the same on a patient without a disability. The success of phase II stemmed from having a PWD panel that provided examples of exemplary and disappointing medical care and PEs. Phase II included a lecture on communication and the ways it may have to change for certain PWDs; the panel of PWDs included in this phase provided students with the opportunity to hear the patient’s perspectives of and preferences in medical care. This part of the session gave an invaluable first-hand account of how the principles taught in this program can profoundly impact future patients.

We expect this program to continue being held annually at FSUCOM. We plan to expand this program to allow for more students to participate and to bring a wider variety of professionals to the COM to speak to students. Furthermore, direct interactions between PWDs and students have been planned for future events. Service dog trainers and handlers, home bedside management technology, and speech-language pathologists are among the stations we intend to include in the future. Additionally, we would like to work with other medical institutions to assist in building similar programs and providing this opportunity to a larger student population. In the long term, we want to work closely with FSUCOM to incorporate a similar program into the medical curriculum as an elective course, a series of shorter required sessions in the regular medical curriculum, or a similar alternative. The ultimate goal of this project is to develop a more robust disability curriculum that provides students with a more in-depth understanding of PWDs and the ways to provide them with optimal care. 

A majority of medical schools do not include disabled standardized patients in their curricula [3]. Studies have shown that only 40.7% of physicians were very confident about their ability to provide the same quality of care to PWDs [4]. To fill this gap, exposure to PWDs in clinical vignettes and PEs should be prioritized to educate future physicians on how to treat all patients regardless of their disability status.

## Conclusions

Disability training in medical academic institutions is a topic that has consistently received insufficient attention. Although there are now more medical and technological advancements than ever to assist PWDs to live longer, fulfilling lives, students and medical professionals still lack the experience and knowledge of interprofessional care required for this patient population. True patient-centered care can require the involvement of a wide variety of interventions, devices, and opinions, yet, we have not been able to identify a dedicated program at any medical institution that encompasses all the aspects of our novel program. Our proposed solution at the FSUCOM began with the DAAP. Although the program is at an infantile stage, it had a significant impact on the FSUCOM student body. Local professionals in physical therapy, occupational therapy, audiology, speech-language pathology, communication, vehicle modification, and motorized wheelchairs were enthusiastic about being able to contribute to this program and expressed a great deal of support. The FSUCOM faculty, medical students, physician assistant students, and the PWD panel also demonstrated overwhelming support for the DAAP. We believe a large portion of the program’s success stemmed from the two-phase interactive model that allowed students to have an immersive learning experience. Informal participant and volunteer feedback supports these conclusions and indicates successful engagement and edification. Future iterations of the program will utilize surveys before and after participation to quantify and analyze changes in student perceptions and knowledge. We believe this analysis will support the assertion that this program has successfully met the intended objectives. In the future, we plan to facilitate more direct interactions between students and PWDs, a deeper discussion on the adaptations involved in the history and PEs for PWDs, and larger participant groups. We hope that this program will provide a framework for essential and needed changes in medical training.
