# Correction: A Novel Disability Advocacy and Awareness Program for Training Future Healthcare Professionals on Care for Patients with Disabilities

**DOI:** 10.7759/cureus.c111

**Published:** 2023-04-25

**Authors:** Priscilla R Morelli, Shemar Crawford, Vivian Tanios, Stephen Chelko, Alexandra Nowakowski

**Affiliations:** 1 Medical Education, Florida State University College of Medicine, Tallahassee, USA; 2 Biomedical Sciences, Florida State University College of Medicine, Tallahassee, USA; 3 Geriatrics, Florida State University College of Medicine, Orlando, USA

This article has been corrected at the request of the authors after discovering that the wrong article was listed as reference 11. Reference 11 has been replaced with the article found here: https://www.sciencedirect.com/science/article/abs/pii/S1936657423000298 In addition, the authors neglected to rightfully acknowledge the authors of this article, as they were previously requested to do. As a result, the following acknowledgement has been added: "We would like to acknowledge Dr. Ami DeWaters and her team for insight and use of their ADEPT-CARE protocol."

